# Coexisting YAP expression and *TP53* missense mutations delineates a molecular scenario unexpectedly associated with better survival outcomes in advanced gastric cancer

**DOI:** 10.1186/s12967-018-1607-3

**Published:** 2018-09-04

**Authors:** Matteo Pallocca, Frauke Goeman, Francesca De Nicola, Elisa Melucci, Francesca Sperati, Irene Terrenato, Laura Pizzuti, Beatrice Casini, Enzo Gallo, Carla Azzurra Amoreo, Patrizia Vici, Luigi Di Lauro, Simonetta Buglioni, Maria Grazia Diodoro, Edoardo Pescarmona, Marco Mazzotta, Maddalena Barba, Maurizio Fanciulli, Ruggero De Maria, Gennaro Ciliberto, Marcello Maugeri-Saccà

**Affiliations:** 1 SAFU Laboratory, Department of Research, Advanced Diagnostic, and Technological Innovation, IRCCS “Regina Elena” National Cancer Institute, Via Elio Chianesi 53, 00144 Rome, Italy; 2 Oncogenomic and Epigenetic Unit, IRCCS “Regina Elena” National Cancer Institute, Via Elio Chianesi 53, 00144 Rome, Italy; 3 Department of Pathology, IRCCS “Regina Elena” National Cancer Institute, Via Elio Chianesi 53, 00144 Rome, Italy; 4 Biostatistics-Scientific Direction, IRCCS “Regina Elena” National Cancer Institute, Via Elio Chianesi 53, 00144 Rome, Italy; 5 Division of Medical Oncology 2, IRCCS “Regina Elena” National Cancer Institute, Via Elio Chianesi 53, 00144 Rome, Italy; 6 Medical Oncology Unit, Policlinico Sant’Andrea, Via Di Grotta Rossa 1035/1039, 00189 Rome, Italy; 7 Institute of General Pathology, Catholic University of the Sacred Heart and Fondazione Policlinico Universitario Agostino Gemelli, Largo Agostino Gemelli, 10, 00168 Rome, Italy; 8 Scientific Direction, IRCCS “Regina Elena” National Cancer Institute, Via Elio Chianesi 53, 00144 Rome, Italy; 9 Division of Medical Oncology 2 and Scientific Direction, IRCCS “Regina Elena” National Cancer Institute, Via Elio Chianesi 53, 00144 Rome, Italy

**Keywords:** Gastric cancer, Hippo pathway, YAP, TP53 mutations

## Abstract

We have previously reported that nuclear expression of the Hippo transducer TAZ in association with Wnt pathway mutations negatively impacts survival outcomes in advanced gastric cancer (GC) patients. Here, we extended these previous findings by investigating another oncogenic cooperation, namely, the interplay between YAP, the TAZ paralogue, and p53. The molecular output of the YAP-p53 cooperation is dependent on *TP53* mutational status. In the absence of mutations, the YAP-p53 crosstalk elicits a pro-apoptotic response, whereas in the presence of *TP53* mutations it activates a pro-proliferative transcriptional program. In order to study this phenomenon, we re-analyzed data from 83 advanced GC patients treated with chemotherapy whose tissue samples had been characterized for YAP expression (immunohistochemistry, IHC) and *TP53* mutations (deep sequencing). In doing so, we generated a molecular model combining nuclear YAP expression in association with *TP53* missense variants (YAP+/TP53^mut(mv)^). Surprisingly, this signature was associated with a decreased risk of disease progression (multivariate Cox for progression-free survival: HR 0.53, 95% CI 0.30–0.91, p = 0.022). The YAP+/TP53^mut(mv)^ model was also associated with better OS in the subgroup of patients who received chemotherapy beyond the first-line setting (multivariate Cox: HR 0.36, 95% CI 0.16–0.81, p = 0.013). Collectively, our findings suggest that the oncogenic cooperation between YAP and mutant p53 may translate into better survival outcomes. This apparent paradox can be explained by the pro-proliferative program triggered by YAP and mutant p53, that supposedly renders cancer cells more vulnerable to cytotoxic therapies.

**Electronic supplementary material:**

The online version of this article (10.1186/s12967-018-1607-3) contains supplementary material, which is available to authorized users.

## Background

Our group recently reported data in the “Journal of Translational Medicine” on the adverse survival outcomes conferred by nuclear expression of the transcriptional co-activator with PDZ-binding motif (TAZ, a downstream effector of the Hippo pathway) in association with Wnt pathway mutations in advanced gastric cancer (GC) [1]. The Hippo pathway is a central regulator of organ development, tissue repair after injury and stem cell fate [2]. The pathway is composed by a regulatory module and a transcriptional module. The first encompasses kinases and adaptors whereas the second is composed by two closely related transcriptional cofactors acting downstream the core regulatory module: TAZ and the Yes-associated protein (YAP). The core module prevents YAP/TAZ nuclear accumulation and interaction with transcriptional partners (e.g. TEAD1–4), refraining YAP/TAZ-driven gene transcription [2]. Deregulation of the Hippo pathway and pervasive activation of YAP/TAZ was observed in a variety of tumors and linked to a number of tumor-promoting activities, spanning from malignant transformation and metastatic dissemination to chemoresistance and self-renewal of cancer stem cells [2]. In GC, YAP knockdown had detrimental effects on cell proliferation, invasion and motility, and the inhibition of the YAP-TEAD interaction hindered tumor growth both in vitro and in vivo [3, 4].

Mutations in central components of the pathway have rarely been detected in cancer genome studies [2]. Thus, a common belief is that deregulation of the Hippo pathway is mostly driven by the dysfunctional nature of stimuli that physiologically fine-tune its activity. Indeed, the Hippo cascade is collocated at the centerpiece of an intricate regulatory network that includes determinants of cell polarity and cell–cell junctions, kinases acting upstream the regulatory module, mechanical forces (mechanotransduction), soluble factors acting through G-protein-coupled receptors (GPCRs) and Rho GTPases, and metabolic routes [2]. Recently, evidence is accumulating conveying the message that the Hippo signaling cooperates with other deregulated pathways in a process that ignites aberrant YAP/TAZ activity. For instance, YAP/TAZ are functionally concatenated with the Wnt pathway whose key components, in turn, are frequently mutated in various tumor types, even including GC [5–7]. Consistently, the expression of TAZ along with Wnt pathway mutations (*APC*, *CTNNB1* and *FBXW7*) negatively impacts survival outcomes in advanced GC patients [1].

Adding further complexity to this picture is the Janus-faced relationship between the Hippo pathway and p53. In a *TP53* wild-type background, YAP and Hippo kinases cooperate with p53 in inducing a tumor suppressive response that culminates into senescence, apoptosis or differentiation [8]. This process is further bolstered by positive feedback loops, given that YAP induces p53 transcription that, in turn, activates YAP and LATS2 transcription. Conversely, in a *TP53* mutated context, YAP and mutant p53 forms a complex that promotes the transcription of key cell cycle regulators including cyclin A, cyclin B, and cyclin-dependent kinase 1 [8]. Thus, the biological output of this transcriptional program is an increase in cellular proliferation.

Here, we investigated the clinical significance of the YAP-p53 partnership in GC, relying on tissue samples from 83 GC patients treated with first-line chemotherapy either in prospective phase II trials or in routine clinical practice. Samples had been already characterized for the expression of a battery of protein- and gene-level biomarkers, also including YAP assessed by immunohistochemistry (IHC) and *TP53* mutations evaluated through targeted DNA next-generation sequencing (NGS) [1, 9]. A detailed description of reagents, procedures and statistical analyses was provided elsewhere [1, 9], whereas baseline characteristics of GC included in this analysis are detailed in the Additional file 1.

Given that the goal of this study was to investigate the clinical significance of YAP-mutant p53-mediated transcription, we generated a molecular model that combined nuclear YAP expression in association with *TP53* missense mutations. Thus, we did not consider positive tumors that harbored other types of mutations (i.e. stop-gain mutations). This is consistent with the documented pattern of *TP53* alterations necessary for interacting with YAP. We referred to this background as the YAP+/*TP53*^mut(mv)^ signature. We did not record any significant association between the YAP+/*TP53*^mut(mv)^ signature and clinical-pathological factors potentially affecting survival outcomes (data available upon request). Moreover, when individually considered, neither YAP expression nor *TP53* mutations were associated with shorter PFS (log-rank p = 0.284 and 0.209, respectively).

### The YAP+/TP53^mut(mv)^ signature is an independent predictor of longer progression-free survival

**Fig. 1:**
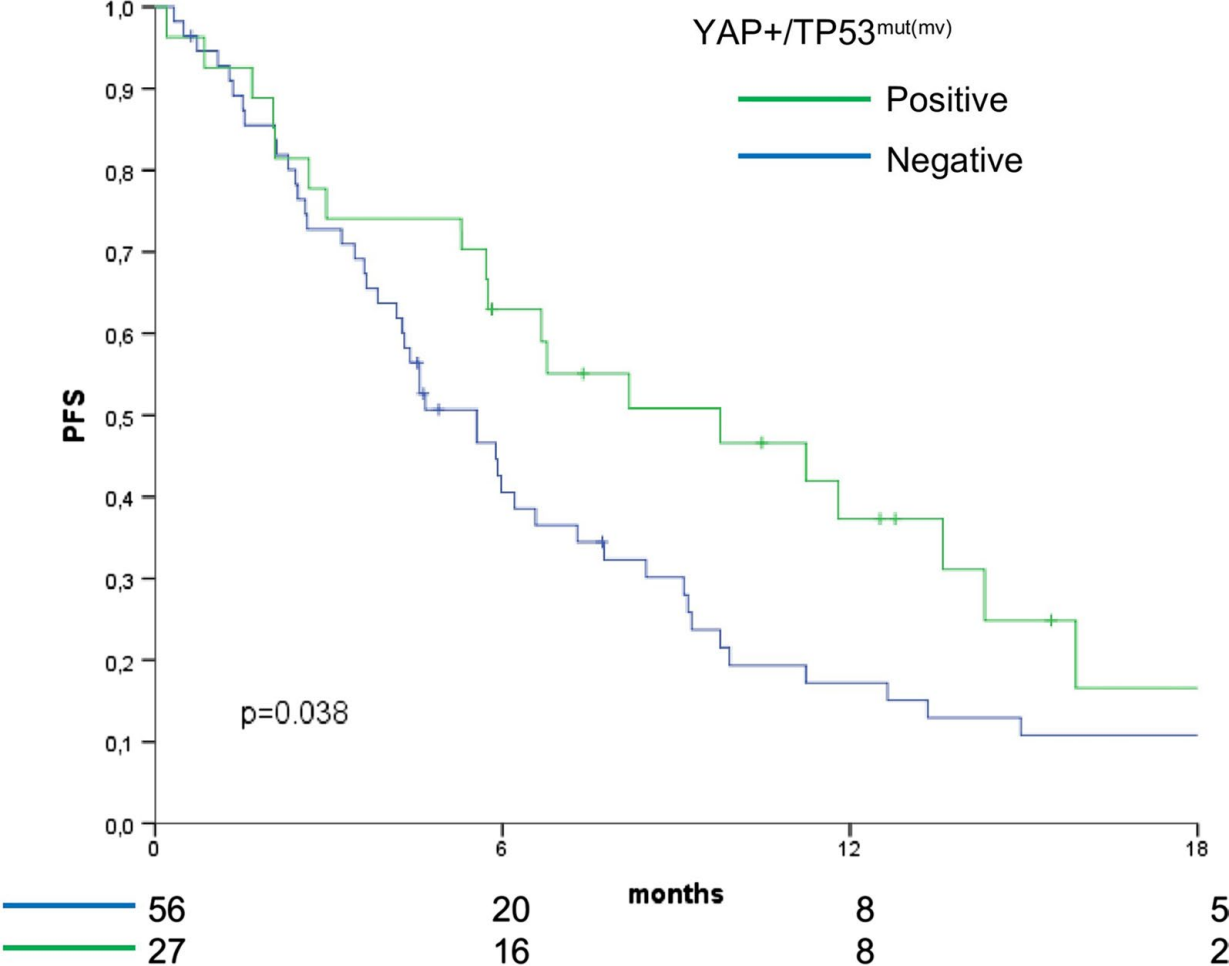
Kaplan–Meier survival curves of progression-free survival comparing YAP+/TP53^mut(mv)^ cases versus their negative counterparts (N = 83)

The individual distribution of YAP expression and *TP53* mutations is presented in Additional file 2, along with the nature of TP53 mutations. Patients whose tumors carried the YAP+/TP53^mut(mv)^ signature had longer PFS compared with their negative counterparts (log rank p = 0.038) (Fig. 1). This latter group was defined as YAP expression without *TP53* missense mutations (wild-type or *TP53* stop-gain mutations) and samples with negative YAP expression, irrespectively of the presence or absence of *TP53* mutations. The protective significance of the YAP+/TP53^mut(mv)^ model was retained in a multivariate Cox regression analysis built by adjusting for other plausible predictors of PFS (HR 0.53, 95% CI 0.30–0.91, p = 0.022) (Table 1). We have previously described two other signatures associated with adverse survival outcomes, the first denoting the TAZ/Wnt pathway crosstalk (TAZ^pos^/WNT^mut)^ and the second indicating the activation of the DNA damage response (DDR) network (γ-H2AX^pos^/pATM^pos^) [1, 9]. Thus, we verified whether the protective role of the YAP+/TP53^mut(mv)^ model was maintained also when taking into account the two aforementioned molecular backgrounds. A molecularly-focused multivariate Cox regression analysis enforced the idea that the YAP+/TP53^mut(mv)^ model holds a protective role (HR: 0.58, 95% CI 0.34–0.99, p = 0.045) independently of other molecular contexts, and also confirmed the robustness of the H2AX^pos^/pATM^pos^ and TAZ^pos^/WNT^mut^ signatures (Additional file 3). Moreover, we did not find any association between the YAP+/*TP53*^mut(mv)^ model and the other two signatures (H2AX^pos^/pATM^pos^ and TAZ^pos^/WNT^mut^ Chi squared test p = 0.759 and p = 0.817, respectively). Internal validation performed through the bootstrap method confirmed the stability of the model.

**Table 1:** Univariate and multivariate Cox regression models for progression-free survival (PFS) (N = 83)

	N (%)	Univariate Cox regression model		Multivariate Cox regression model	
		HR (95% CI)	p-value	HR (95% CI)	p-value
YAP+/TP53mut^(mv)^	Positive vs negative 27 (32.5%) vs 56 (67.5%)	0.58 (0.34–0.98)	0.040	0.53 (0.30–0.91)	0.022
ECOG-PS	1–2 vs 0 39 (47.0%) vs 44 (53.0%)	1.27 (0.79–2.04)	0.325	1.21 (0.74–1.98)	0.439
Stage	Metastatic vs locally advanced 47 (56.6%) vs 36 (43.4%)	1.24 (0.77–1.99)	0.384	1.48 (0.84–2.59)	0.171
Localization	Stomach vs EOJ 76 (91.6%) vs 7 (8.4%)	0.68 (0.29–1.59)	0.376	0.61 (0.23–1.61)	0.317
No. metastatic sites	2–3 vs 1 25 (30.1%) vs 58 (69.9%)	1.41 (0.83–2.37)	0.199	1.20 (0.67–2.15)	0.541
Taxanes	Yes vs no 44 (53.0%) vs 39 (47.0%)	0.81 (0.50–1.31)	0.395	0.73 (0.42–1.28)	0.274

### The impact of the YAP+/TP53^mut(mv)^ signature on overall survival is dependent on post-first-line chemotherapy

**Fig. 2:**
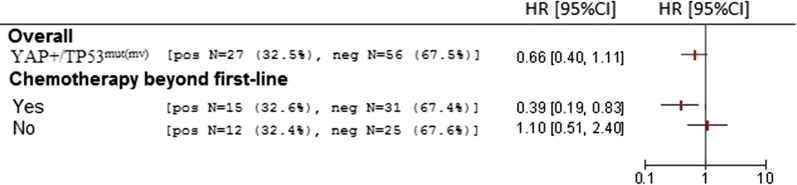
Forest plot illustrating univariate Cox regression analyses (YAP+/TP53^mut(mv)^) for overall survival. From top to bottom: entire cohort, subgroup of patients who received chemotherapy beyond the first-line, subgroup of patients who did not receive second-line chemotherapy

We finally verified whether the YAP+/TP53^mut(mv)^ model was associated with better OS. Unexpectedly, we did not record any significant association in the entire cohort (univariate Cox for OS: HR 0.66, 95% CI 0.44–1.11) (Fig. 2). Prompted by this observation, we reasoned that the connection between YAP+/TP53^mut(mv)^ and OS might be tied to the administration of chemotherapy beyond the first-line setting. This hypothesis is rooted in the transcriptional output of the YAP-mutant p53 complex, which up-regulates various cell cycle controllers and culminates into increased cellular proliferation. Consistently, we observed that Ki-67 levels, assessed in a subgroup of samples, were significantly higher in YAP+/TP53^mut(mv)^-positive vs negative cases (p = 0.031) (Additional file 4). On this premise, we tested the subgroup of patients who received second-line chemotherapy, and the subset of patients who did not receive chemotherapy beyond the first-line setting. With this approach, we noticed that the YAP+/TP53^mut(mv)^ signature was associated with a decreased risk of death exclusively in patients who received second-line (and eventually subsequent) chemotherapy (HR 0.39, 95% CI 0.19–0.83) (Fig. 2). This observation was confirmed in a multivariate Cox regression analysis (HR: 0.36, 95% CI 0.16–0.81, p = 0.013) (Table 2). Collectively, these findings corroborate the idea that the YAP+/TP53^mut(mv)^ signature favorably impacts survival outcomes, and that its protective role is linked to the administration of chemotherapy.

**Table 2:** Univariate and multivariate Cox regression models for overall survival (OS) in patients who received chemotherapy beyond the first-line setting (N = 46)

	N (%)	Univariate Cox regression model		Multivariate Cox regression model	
		HR (95% CI)	p-value	HR (95% CI)	p-value
YAP+/TP53mut^(mv)^	Positive vs negative 15 (32.6%) vs 31 (67.4%)	0.39 (0.19–0.83)	0.015	0.36 (0.16–0.81)	0.013
ECOG-PS	1–2 vs 0 19 (41.3%) vs 27 (58.7%)	1.17 (0.61–2.23)	0.636	1.61 (0.77–3.39)	0.209
Stage	Metastatic vs locally advanced 24 (52.2%) vs 22 (47.8%)	1.21 (0.63–2.31)	0.564	1.44 (0.60–3.47)	0.414
No. metastatic sites	2–3 vs 1 16 (34.8%) vs 30 (65.2%)	1.16 (0.60–2.24)	0.666	0.68 (0.30–1.54)	0.355
Taxanes	Yes vs no 25 (54.3%) vs 21 (45.7%)	0.77 (0.40–1.48)	0.440	0.72 (0.30–1.68)	0.445

## Discussion

This study capitalizes on a growing body of evidence that assigns oncogenic functions to the YAP-p53 cooperation [8]. In order to address the clinical relevance of such a vicious interaction, we evaluated a relatively large series of advanced GC patients treated with chemotherapy. Approximately half of the patients examined were enrolled in prospective phase II trials. Our results suggested that a molecular background registering YAP-p53-driven transcription, and consequently accelerated neoplastic proliferation, is associated with better survival outcomes. The link between the YAP+/*TP53*^mut(mv)^ signature and clinical outcomes emerged when we exclusively considered oncogenic *TP53* missense variants. This is consistent with a mutational pattern necessary to encode a protein able to interact with YAP. It is worth mentioning that in a molecularly-focused multivariate Cox regression analysis, the YAP+/*TP53*^mut(mv)^ model was associated with a decreased risk of disease progression independently from the two other signatures we had previously identified [1, 9]. The observation that the three signatures retained their predictive value enables us to exclude potential confounding factors.

To our knowledge, this is the first report striving to address how two cooperating oncogenic forces, namely YAP and mutant p53, impact survival outcomes in advanced GC patients. The seemingly paradoxical finding that the YAP+/*TP53*^mut(mv)^ signature is associated with better survival outcomes in chemotherapy-treated GC raises some interesting points. First, even though YAP is an established tumor-promoting factor, in some instances tumor-suppressive functions have been described [10]. For instance, (i) YAP interacts with, and stabilizes, p73 leading to transcription of pro-apoptotic target genes, (ii) as aforementioned, in the absence of *TP53* mutations the crosstalk between YAP and p53 leads to the induction of cell death stimuli, (iii) YAP is negatively regulated by oncogenic AKT, and (iv) in some cases such as breast cancer, multiple evidence side with a tumor suppressive function of YAP, including both frequent loss of heterozygosity at 11q22.2 and mechanistic studies [10]. Thus, while YAP is widely perceived as an oncogene, in some contexts it seems to be endowed with tumor-suppressive capabilities. In GC, in vitro and in vivo experiments showed that YAP boosts tumor progression [3, 4], whereas our data point to YAP as a biomarker of greater efficacy of chemotherapy in a *TP53*-dependent manner. The explanation of such inconsistency may be fairly intuitive. The YAP-mutant p53 transcriptional program accelerates cellular proliferation by up-regulating master regulators of the cell cycle; this, in turn, should increase chemosensitivity. Consistently with this hypothesis, the relationship between the YAP+/*TP53*^mut(mv)^ model and OS was strictly dependent on the administration of chemotherapy. Indeed, OS analyses revealed that the YAP+/*TP53*^mut(mv)^ signature was associated with better OS exclusively in the subset of patients who received second-line chemotherapy, whereas this relationship was lost in the subgroup of patients who were not treated with chemotherapy beyond the first-line setting. Thus, the model we propose is that an oncogenic process fueling the proliferation of neoplastic cells turns into a vulnerability trait upon exposure to chemotherapy. The observation that tumors carrying the YAP+/TP53^mut(mv)^ signature have higher Ki67 levels is consistent with our hypothesis. It is worth mentioning that we were unable to perform comparable analyses in publically available datasets (e.g. TCGA). Indeed, other independent databases do not contain the necessary information to verify our findings including PFS, extensive data on administered chemotherapy throughout the natural history of the disease, and YAP expression/localization at the protein level (to our knowledge, RPPA data in the TCGA contains the phosphorylated, and therefore inactive, form of YAP). Nevertheless, the stability of our signature was confirmed with the bootstrap method, representing the most accurate procedure for internal validation.

Another aspect that needs to be considered is that the interaction between the Hippo pathway and p53 extends beyond the molecular frame herein investigated [8]. For instance, p53 also cooperates with the Hippo kinases. LATS2 stabilizes p53, whereas the silencing of LATS1 and LATS2 modifies p53’ conformation and interactome, shifting p53 function toward a mutant-like state that promotes cell migration. Beyond p53, YAP also associates with p73 leading to the transcription of pro-apoptotic target genes such as *PUMA* and *p53AIP1*. Taking into account the multi-level interaction between the Hippo signaling cascade and p53 family members [8], in our opinion a deeper molecular characterization is needed to fully appreciate the clinical implications of such cooperation. To address this issue, we designed a prospective study with biomarker validation purposes on the basis of our previous results [1, 9]. The trial envisions an extensive genomic and transcriptomic analysis that will enable us to carry out an integrated pathway-focused analysis. This will be instrumental to achieve a deeper understanding of this process.

Finally, the molecular characterization of GC revealed the existence of four molecular subtypes: chromosomal instability (CIN), microsatellite instability (MSI), genomically stable (GS) and Epstein–Barr virus (EBV)-positive [7]. Deregulation of the Hippo pathway in GS-GC is suggested by *RHOA* and *CDH1* mutations, along with *CLDN18*–*ARHGAP26* fusions [7]. All these alterations impact established regulatory branches of the Hippo pathway such as Rho GTPases, cell–cell adhesion mechanisms and cell polarity factors. Moreover, EBV-related GC is characterized by DNA hypermethylation, and both *MOB1B* and *WWTR1,* the gene encoding for TAZ, present frequent promoter hypermethylation [7]. Thus, the molecular taxonomy of GC adds further complexity to a yet intricate phenomenon, raising the idea that individual molecular subtype deserves increased attention in studies striving to assess the clinical exploitability of the Hippo-p53 communication.

## Conclusions

Collectively, our data suggest that the YAP-mutant p53 interaction denotes a subset of advanced GC with better survival outcomes. Our hypothesis is that this unexpected association is rooted in a pro-proliferative program triggered by YAP and mutant p53 proteins, that render cancer cells more susceptible to chemotherapy-induced death stimuli. Wider molecular analyses within the context of prospective studies are warranted to provide further evidence on how this information can be transferred to the clinical setting.

## Additional files

**Additional file 1.** Baseline characteristics of gastric cancer (GC) patients included in this study (N = 83).

**Additional file 2.** Panel A) Oncoprint illustrating the individual distribution of YAP expression (positive and negative) and *TP53* mutations; panel B) Mutation Mapper illustrating the detected mutations represented on the linear protein. Missense variants (green) have been considered for building the YAP+/TP53^mut(mv)^ model.

**Additional file 3.** Multivariate Cox regression model for progression-free survival (PFS) evaluating three different molecular signatures (N = 83).

**Additional file 4.** Box plot showing the distribution of Ki67 values in the YAP+/Tp53^mu t(mv)^ positive and negative group (N = 23; YAP+/TP53^mut(mv)^-positive cases N = l 4; YAP+/TP53^mut(mv)^-negative cases N = 9). The percentage of positive cells was calculated considering approximately 2000 tumor cells from four randomly selected high-power fields (×400).

## Abbreviations

GC: gastric cancer
IHC: immunohistochemistry
NGS: next-generation sequencing
OS: overall survival
PFS: progression-free survival
TAZ: transcriptional co-activator with PDZ-binding motif
YAP: yes-associated protein
