# Supplementary material for: Coexisting YAP expression and TP53 missense mutations delineates a molecular scenario unexpectedly associated with better survival outcomes in advanced gastric cancer
Source: J Transl Med. 2018 Sep 4;16:247. doi: 10.1186/s12967-018-1607-3 (PMC6122687; doi:10.1186/s12967-018-1607-3)
Supplement: Supplementary file 1 — Additional file 1. Baseline characteristics of gastric cancer (GC) patients included in this study (N = 83). [file 12967_2018_1607_MOESM1_ESM.doc]

Additional File 1: Baseline characteristics of gastric cancer (GC) patients included in this study (N=83).

| **Characteristics** | **N (%)** |
| --- | --- |
| **Age at diagnosis** Median (min-max) [IQ range] | 60.8 (28-79) [53.2-67.4] |
| **Gender** |  |
| Male | 43 (51.8) |
| Female | 40 (48.2) |
| **ECOG PS** |  |
| 0 | 44 (53.0) |
| 1-2 | 39 (47.0) |
| **Stage** |  |
| Locally advanced | 36 (43.4) |
| Metastatic | 47 (56.6) |
| **Previous surgery** |  |
| No | 28 (33.7) |
| Yes | 55 (66.3) |
| **Neoadjuvant/adjuvant chemotherapy** |  |
| No | 57 (68.7) |
| Yes | 26 (31.3) |
| **Lauren classification** |  |
| Intestinal | 34 (41.0) |
| Diffuse | 40 (48.2) |
| Mixed | 9 (10.8) |
| **Grade** |  |
| G2 | 19 (22.9) |
| G3 | 63 (75.9) |
| Unknown | 1 (1.2) |
| **Localization** |  |
| Esophagogastric junction (EOJ) | 7 (8.4) |
| Stomach | 76 (91.6) |
| **Agents** |  |
| 2 | 36 (43.4) |
| 3 | 47 (56.6) |
| **Taxanes** |  |
| No | 39 (47.0) |
| Yes | 44 (53.0) |
| **Chemotherapy beyond the first-line** |  |
| No | 37 (44.6) |
| Yes | 46 (55.4) |
