# Supplementary figures and images for: Coexisting YAP expression and TP53 missense mutations delineates a molecular scenario unexpectedly associated with better survival outcomes in advanced gastric cancer
Source: J Transl Med. 2018 Sep 4;16:247. doi: 10.1186/s12967-018-1607-3 (PMC6122687; doi:10.1186/s12967-018-1607-3)

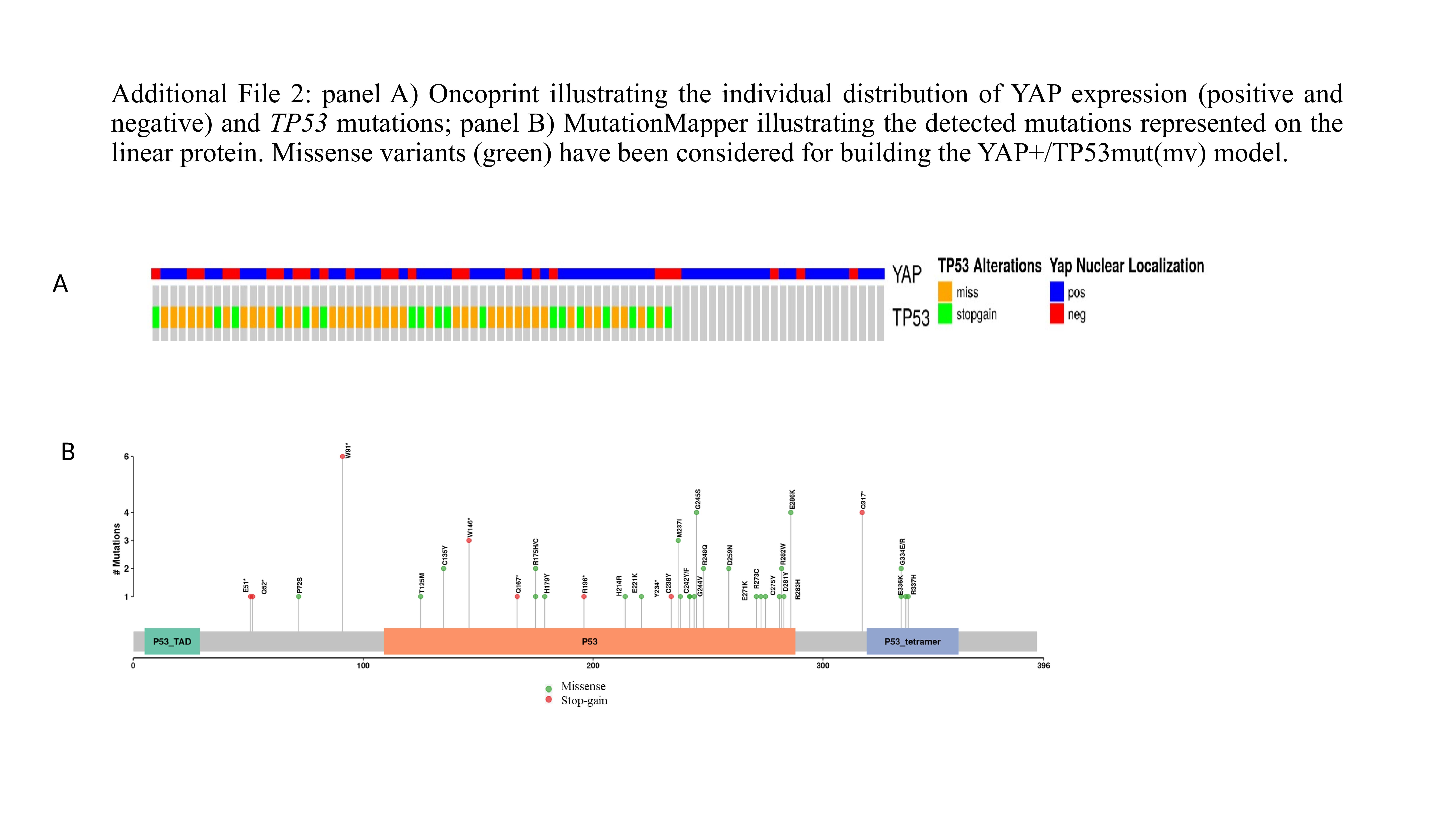

Supplement: Supplementary file 2 — Additional file 2. Panel A) Oncoprint illustrating the individual distribution of YAP expression (positive and negative) and TP53 mutations; panel B) Mutation Mapper illustrating the detected mutations represented on the linear protein. Missense variants (green) have been considered for building the YAP+/TP53mut(mv) model. [file 12967_2018_1607_MOESM2_ESM.jpg]

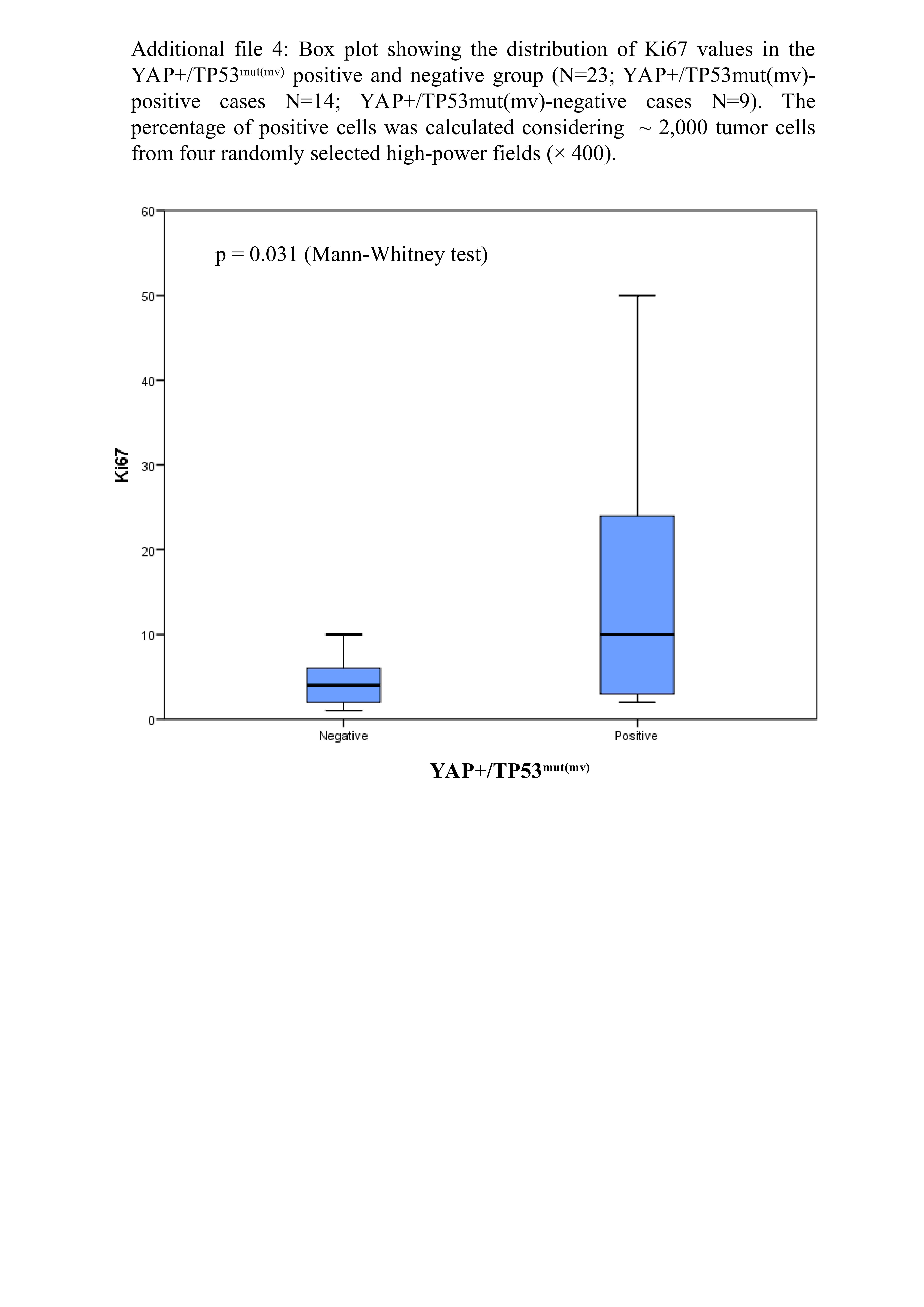

Supplement: Supplementary file 4 — Additional file 4. Box plot showing the distribution of Ki67 values in the YAP+/Tp53mu t(mv) positive and negative group (N = 23; YAP+/TP53mut(mv)-positive cases N = l 4; YAP+/TP53mut(mv)-negative cases N = 9). The percentage of positive cells was calculated considering approximately 2000 tumor cells from four randomly selected high-power fields (×400). [file 12967_2018_1607_MOESM4_ESM.jpg]
