# Supplementary material for: Coexisting YAP expression and TP53 missense mutations delineates a molecular scenario unexpectedly associated with better survival outcomes in advanced gastric cancer
Source: J Transl Med. 2018 Sep 4;16:247. doi: 10.1186/s12967-018-1607-3 (PMC6122687; doi:10.1186/s12967-018-1607-3)
Supplement: Supplementary file 3 — Additional file 3. Multivariate Cox regression model for progression-free survival (PFS) evaluating three different molecular signatures (N = 83). [file 12967_2018_1607_MOESM3_ESM.doc]

Additional File 3: Multivariate Cox regression model for progression-free survival (PFS) evaluating three different molecular signatures (N=83).

|  |  | **Multivariate Cox**  **regression model** | |
| --- | --- | --- | --- |
|  |  | **HR (95%CI)** | **p-value** |
| **YAP+/TP53^mut(mv)^** | **Positive vs Negative** | **0.58 (0.34-0.99)** | **0.045** |
| **γH2AX^pos^/pATM^pos^** | **Positive vs Negative** | 2.66 (1.55-4.57) | <0.001 |
| **TAZ^pos^/WNT^mut^** | **Positive vs Negative** | 1.92 (1.06-3.46) | 0.031 |
